# Identifying relevant topics and their competency levels for dental materials science: a fuzzy Delphi study

**DOI:** 10.1186/s12903-023-02946-8

**Published:** 2023-04-27

**Authors:** Galvin Sim Siang Lin, Kah Jun Pow, Noor Azlin Yahya, Chan Choong Foong, Tahir Yusuf Noorani

**Affiliations:** 1 Department of Dental Materials, Faculty of Dentistry, Asian Institute of Medicine, Science and Technology (AIMST) University, Bedong, 08100 Kedah Malaysia; 2 Pulau Pinang State Health Department, Ministry of Health Malaysia, 10590 Pulau Pinang, Malaysia; 3 Department of Restorative Dentistry, Faculty of Dentistry, Universiti Kebangsaan Malaysia, 50300 Kuala Lumpur, Malaysia; 4 Department of Restorative Dentistry, Faculty of Dentistry, Universiti Malaya, 50603 Kuala Lumpur, Malaysia; 5 Medical Education and Research Development Unit (MERDU), Faculty of Medicine, Universiti Malaya, 50603 Kuala Lumpur, Malaysia; 6 Conservative Dentistry Unit, School of Dental Sciences, Universiti Sains Malaysia, Health Campus, 16150 Kubang Kerian, Kelantan Malaysia

**Keywords:** Curriculum development, Delphi technique, Dental education, Dental materials, Undergraduate

## Abstract

**Background:**

Dental materials science is an important core course in undergraduate dental programs which integrates foundational concepts of chemical engineering and materials science into clinical dentistry. The present study aimed to identify relevant dental materials science topics for Malaysian undergraduate dental curricula and to determine their appropriate competency levels in terms of cognitive and psychomotor taxonomies.

**Methods:**

Potential dental materials science topics were drafted in alignment with the revised national competency statement. The list of topics was further amended after comparing it with those recommended topics in the literature. Fuzzy Delphi method was applied. Experts were selected based on the different inclusion criteria. They ranked the topics using a five-point Likert scale and recommended the appropriate cognitive and psychomotor levels. Next, fuzzy evaluation was performed. Consensus was deemed for a topic to be included if (a) the average expert agreement was ≥ 75%, (b) the d-construct threshold value for each topic was ≤ 0.2 and (c) the average fuzzy number was ≥ 0.5.

**Results:**

Sixty-two experts participated in the study. They accepted 33 out of 36 potential dental materials science topics. The average Likert score and fuzzy number ranged from 3.63 to 4.92 and 0.526 to 0.784, respectively. Furthermore, “Endodontic materials” was ranked as the most significant topic. Meanwhile, many topics required dental students to demonstrate a cognitive level of “Apply” and a psychomotor level of “Guided response”. Based on mean scores, “Impression materials” was rated as the most cognitively demanding topic, whilst “Temporary restorative materials” was the most demanding topic for psychomotor taxonomy.

**Conclusion:**

The present study has identified relevant dental materials science topics and their appropriate cognitive and psychomotor levels using the Fuzzy Delphi approach. The findings of the present study form the basis for future studies to develop measurable learning outcomes, design corresponding innovative pedagogy and propose assessment criteria for each topic.

## Background

The first dental school in Malaysia was established in 1971 and the number has escalated to 13 schools (six public and seven private) in 2023 to serve the demand of rising population [1]. Competency-based education is mandated by the national accreditation agency and requires each dental school to adopt Bloom’s (cognitive) and Simpson’s (psychomotor) taxonomies as the competency framework for designing learning objectives and educational goals. Bloom’s Taxonomy, developed by Benjamin Bloom and his colleagues in the 1950s [2], is a hierarchical framework that identifies six levels of cognitive complexity involved in learning [3]. The levels in ascending order of complexity are: C1 - Remember, C2 - Understand, C3 - Apply, C4 - Analyse, C5 - Evaluate, and C6 - Create. On the other hand, Simpson’s Taxonomy which was developed by E.J. Simpson, in the 1970s, is a similar framework that focuses on the complexity of psychomotor skills. Simpson’s taxonomy identifies seven levels of complexity involved in motor skills, ranging from simple movements to complex integrated movements [4]. The levels in ascending order of complexity are: P1 - Perception, P2 - Set, P3 - Guided response, P4 - Mechanism, P5 - Complex overt response, P6 - Adaptation, P7 - Origination.

In addition, Malaysian dental programs are undergraduate entries and delivered in English. They are organized into preclinical and clinical phases. Dental students learn basic medical and dental sciences during the preclinical phase for two years, and subsequently, they progress to provide patient care in clinics under supervision in the clinical phase for three years [5]. Various courses (topics) are designed to nurture the competencies of dental students. Dental materials science is an important core course in Malaysian dental programs which integrates foundational concepts of chemical engineering and materials science into clinical dentistry [6]. The course aims to provide a thorough understanding of the compositions, characteristics, properties, and manipulation of materials frequently used in dental clinics and laboratories. Over the past 100 years, the field of dental materials science has witnessed a vast revolution with new biomaterials being developed and marketed at an astounding rate [7]. Hence, dental schools need to regularly evaluate dental materials science curricula to keep abreast with the rapid evolution of knowledge in the field and to equip future dentists with the desired knowledge and skills.

Recently, the Malaysian Dental Council (MDC) published a revised competency statement for dental graduates [8]. By the end of dental programs, in terms of mastery in dental materials science, dental graduates should be able to justify the selection of dental materials based on the science and applications (Bloom’s cognitive level: C4 Analyse), and manipulate commonly used dental materials (Simpson’s psychomotor level: P5 Complex overt response). Consequently, dental schools are urged to revamp their existing dental materials science curriculum. However, Malaysian dental schools operate independently without a standardized national curriculum. Due to the absence of a national standard, dental material science curricula vary substantially across different dental schools. Inconsistencies in undergraduate training on dental materials science may have an impact on newly graduated dental practitioners as dental materials are the cornerstone of their daily dental practice. Undoubtedly, dental students who are incompetent in selecting and manipulating commonly used dental materials might result in subpar dental services.

The revised competency statement represents a national advocacy, and hence identifying appropriate curriculum content would be the first step in harmonizing a national curriculum for dental materials science courses. It is a challenging task because each dental school is historically empowered to design and implement their own curriculum. An ideal solution is to build consensus by considering different inputs and perspectives, and the Delphi method is deemed a reliable approach with minimal bias [9–11]. Several studies have employed the Delphi method, notably those in the field of dentistry, such as developing curricula for periodontics and orthodontics [9, 11, 12], general dental practitioners’ competencies [10], and clinical practice guidelines on antibiotic prophylaxis [13]. Sequential surveys are answered (in isolation) by experts to indicate the likelihood of potential occurrences in the area of interest [9]. A modified Delphi version, Fuzzy Delphi method, which is a cost- and time-effective method, can systematically measure and report consensus through generating fuzzy numbers based on respondents’ answers using cumulative frequency distributions and fuzzy scores [14]. Therefore, the present study aimed to identify relevant topics and their competency levels for dental materials science course using Fuzzy Delphi method.

## Methods

The present study was approved by the Asian Institute of Medicine, Science and Technology (AIMST) University Human Ethic Committee (AUHEC) (Approval number: AUHEC/FOD/2022/37).

### Development of the list of relevant topics

A dental materials expert and two restorative dental specialists working at different public and private institutions formed a team to draft topics for dental materials science for undergraduate dental education. First, they reviewed the existing curriculum in each dental school. Topics which correspond to the revised national competency statement were identified. Subsequently, using keywords “dental materials”, “dental biomaterials” and “materials in dentistry”, they searched for related articles in Google Scholar, PubMed, Web of Science and Scopus databases. The search was restricted to English-language review articles on dental materials science published between January 2000 and November 2022. Each article abstract was screened, and they were excluded if the articles did not contain content in dental materials or materials used in dentistry. As a result, eleven review articles were selected after duplications were removed. Based on the institutional existing curriculum and selected review articles, relevant topics for dental materials science were identified and discussed among the members. The list of relevant topics was revised and finalized until a consensus was achieved. The draft consisted of a list of 36 relevant topics which was converted into a Google Form questionnaire to be readily used in the Fuzzy Delphi method.

### Delphi participants

The Delphi method with fuzzy theory was used to measure and report opinions among different experts [15]. The Google Form questionnaire was distributed to a purposive sample comprising experts from various geographical regions of Malaysia [16]. Inclusion criteria for experts were having a basic dental degree with a valid annual/ temporary practising certificate, and fulfilling one of the following criteria: (a) dental specialist with a postgraduate qualification in conservative dentistry, restorative dentistry, prosthodontics, or endodontics, or (b) full-time faculty member with a postgraduate qualification in dental materials science (dental materials expert), or (c) full-time faculty member who has been involved with teaching and research in dental materials science; or (d) general dental practitioner with special interest in dental materials science who is currently enrolled in any postgraduate training program related to conservative dentistry, restorative dentistry, prosthodontics, endodontics, or dental materials science / published in a peer-reviewed journal / given a professional presentation / are actively involved in an organization that upholds the best interests of dental materials science education [11, 12].

### Fuzzy Delphi method

The importance of each topic was determined by the experts anonymously and in isolation, without imposing ideas on each other. Each expert was required to rank each item (topic) based on the five-point Likert scales (1 = Not important at all, 2 = Not important, 3 = Less important, 4 = Important, 5 = Very important), and to state the appropriate competency level for each topic based on Bloom’s cognitive (1 = remember, 2 = understand, 3 = apply, 4 = analyse, 5 = evaluate, 6 = create) and Simpson’s psychomotor (1 = perception, 2 = set, 3 = guided response, 4 = mechanism, 5 = complex overt response, 6 = adaptation, 7 = origination) taxonomies. Experts were allowed to add topics if they were not in the list.

The process was followed by converting linguistic variables to fuzzy numbers. There were three numbers for each recorded response to consider which are the average minimum value, the most reasonable value, and the maximum value. In other words, a triangular fuzzy number is symbolized as a triangle and contains the values of *m*1, *m*2, and *m*3 [17]. *m*1 represents the lowest value, *m*2 is a fair value, and *m*3 represents the highest value. The m values showed the probability that the experts agreed that the dental materials science topics were important. For instance, a Likert scale of 3 indicates that the *m*1 value shows at least 20% of the experts agree it was important, *m*2 represents a reasonably average likelihood that 40% of the experts would agree the topic was important, while *m*3 signifies that at most 60% of experts would agree that the topic was important.

The defuzzification procedure involved ranking each item (topic) to determine its level of relevance and subsequently decide to keep or to remove the topic. Three criteria were applied. First, to determine the acceptability of the topics, the experts were deemed to reach a consensus if the difference between the average and expert evaluation data was less than or equal to the threshold value of 0.2 [18]. The *d*-construct threshold value for each item was identified by determining the difference between each expert fuzzy number and the average fuzzy number. The vertex technique was applied to determine the distance between the average [19]. Second, it was regarded as acceptable if the experts reached a consensus of ≥ 75%. Average experts’ agreement which is less than 75% led to the discarding of certain items and a subsequent round of surveys might be required. Third, a topic with an average fuzzy number ≥ 0.5 was accepted. Then, Fuzzy assessments were performed to model a framework of curriculum content in dental materials science. This phase involved ranking the topics based on consensus of the experts with the highest value being determined by the importance level of each topic in the model [17].

### Cognitive and psychomotor levels analysis

Each competency level in Bloom’s cognitive and Simpson’s psychomotor taxonomies was given a value from 1 to 6, and 1 to 7 respectively. Experts’ responses for each topic were calculated to obtain a mean score; missing responses were excluded [11]. Mean scores determined desired cognitive and psychomotor levels for a particular topic.

## Results

**Fig. 1:**
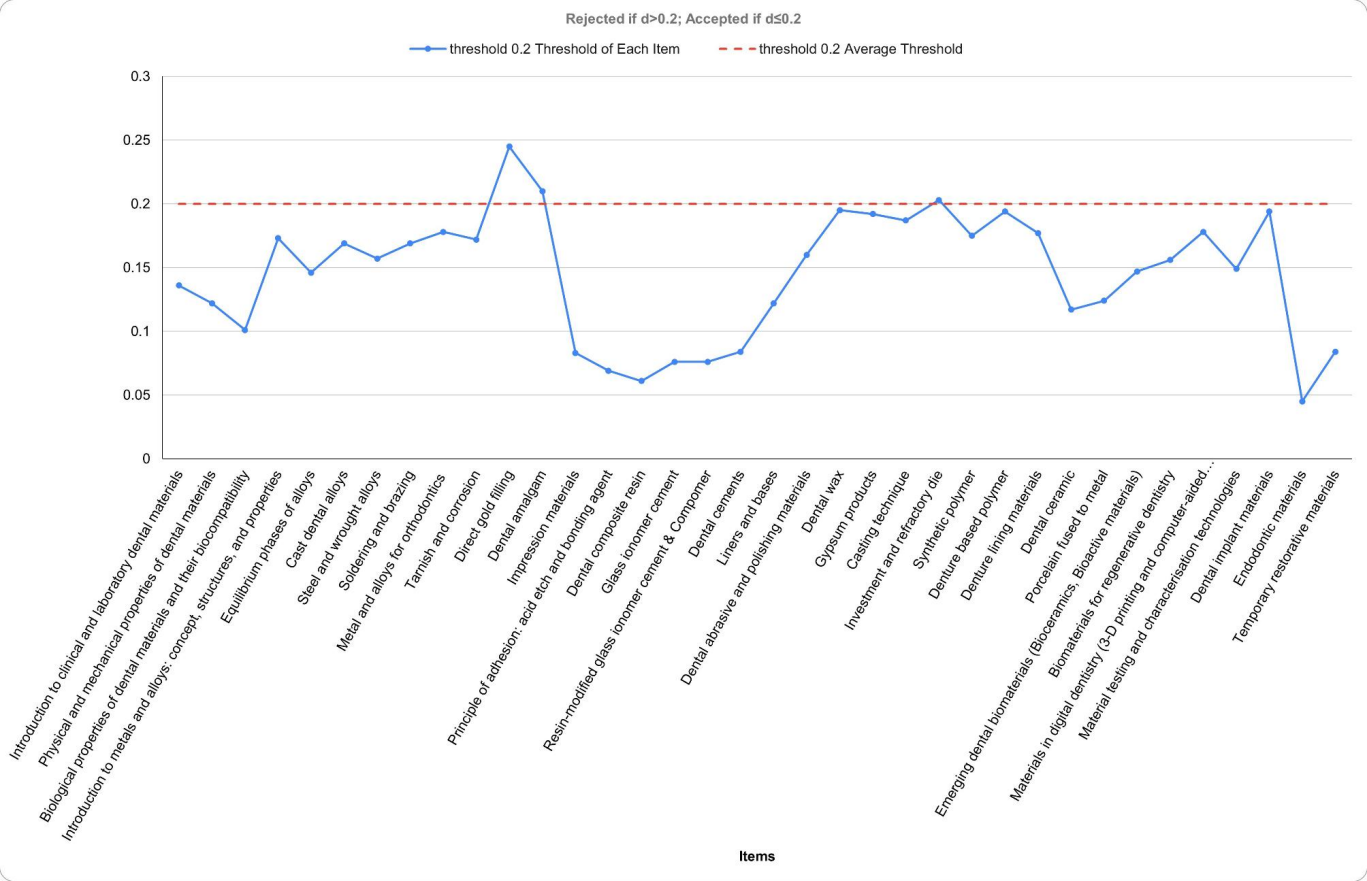
Rejected and accepted dental materials science topics based on the threshold value *d*

The questionnaire was distributed to sixty-five experts who met the inclusion criteria but only sixty-two of them agreed and consented to participate in the questionnaire survey. Their demographic backgrounds are listed in Table 1. Most of the experts had more than 10 years of experience in related fields and were affiliated with public universities in Malaysia. The expert panel arrived at a consensus that 33 out of 36 topics were relevant to the undergraduate dental materials science curriculum (Table 2). The average Likert score ranged from 3.63 to 4.92, while the average fuzzy number for the topics ranged from 0.526 to 0.784. Furthermore, the topic “Endodontic materials” was ranked as the most significant topic, followed by “Dental composite resin” and “Principle of adhesion: acid etch and bonding agent” which corresponds to the second and third most significant topics, respectively. Nonetheless, three topics, “Direct gold filling”, “Dental amalgam”, and “Investment and refractory die”, were removed due to a lack of consensus with *d*-value ˃2 and expert agreement of < 75% (Fig. 1). No additional topics were suggested.

**Table 1:** Demographic backgrounds of the recruited fuzzy Delphi respondents

Items	respondent (*n*)
Field of Expertise	
Restorative dental specialist	16
Prosthodontist	11
Endodontist	11
Dental materials expert	2
General dental practitioner undergoing postgraduate training	22
*Total*	62
Years of experience	
Less than 5 years	9
5 to 10 years	24
More than 10 years	29
*Total*	62
Affiliation	
Public teaching institution	29
Private teaching institution	11
Ministry of Health public hospital/clinic	16
Private hospital/clinic	6
*Total*	62

**Table 2:** Fuzzy Delphi findings on topics and their competency levels for dental materials science

No	Topic	Average Likert score	Threshold value *d* ≤ 0.2	Expert’s Agreement (%)	Average fuzzy number	Ranking	Verdict	Bloom’s Cognitive Level		Simpson’s Psychomotor Level	
								**Mean score**	**Corresponding level**	**Mean score**	**Corresponding level**
1	Introduction to clinical and laboratory dental materials	4.69	0.136	75	0.739	14	Accept	3.03	Apply	3.24	Guided response
2	Physical and mechanical properties of dental materials	4.73	0.122	75	0.745	12	Accept	3.42	Apply	3.40	Guided response
3	Biological properties of dental materials and their biocompatibility	4.79	0.101	79	0.758	9	Accept	3.63	Apply	3.52	Guided response
4	Introduction to metals and alloys: concept, structures, and properties	4.47	0.173	95	0.694	20	Accept	2.95	Understand	2.85	Set
5	Equilibrium phases of alloys	3.92	0.146	85	0.597	32	Accept	2.52	Understand	2.65	Set
6	Cast dental alloys	4.16	0.169	82	0.668	27	Accept	2.79	Understand	2.85	Set
7	Steel and wrought alloys	4.03	0.157	82	0.613	30	Accept	2.76	Understand	2.92	Set
8	Soldering and brazing	3.76	0.169	76	0.552	35	Accept	2.55	Understand	2.81	Set
9	Metal and alloys for orthodontics	4.02	0.178	80	0.732	15	Accept	2.84	Understand	2.97	Set
10	Tarnish and corrosion	4.35	0.172	92	0.671	26	Accept	3.03	Apply	2.97	Set
11	Direct gold filling	3.63	0.245	69	0.526	36	Reject	2.82	Understand	2.85	Set
12	Dental amalgam	3.94	0.21	34	0.594	33	Reject	3.58	Apply	4.08	Mechanism
13	Impression materials	4.84	0.083	84	0.768	8	Accept	4.29	Analyse	4.34	Mechanism
14	Principle of adhesion: acid etch and bonding agent	4.87	0.069	87	0.774	3	Accept	4.23	Analyse	4.26	Mechanism
15	Dental composite resin	4.89	0.061	89	0.777	2	Accept	4.26	Analyse	4.39	Mechanism
16	Glass ionomer cement	4.85	0.076	85	0.771	4	Accept	4.18	Analyse	4.37	Mechanism
17	Resin-modified glass ionomer cement & Compomer	4.85	0.076	85	0.771	4	Accept	4.18	Analyse	4.35	Mechanism
18	Dental cements	4.84	0.084	85	0.768	6	Accept	4.24	Analyse	4.31	Mechanism
19	Liners and bases	4.74	0.122	77	0.748	11	Accept	4.10	Analyse	4.31	Mechanism
20	Dental abrasive and polishing materials	4.61	0.16	95	0.723	17	Accept	3.87	Apply	4.13	Mechanism
21	Dental wax	4.37	0.195	90	0.687	23	Accept	3.47	Apply	3.84	Guided response
22	Gypsum products	4.34	0.192	87	0.674	25	Accept	3.45	Apply	3.87	Guided response
23	Casting technique	4.00	0.187	75	0.600	31	Accept	2.92	Understand	3.31	Guided response
24	Investment and refractory die	3.84	0.203	44	0.568	34	Reject	2.81	Understand	3.26	Guided response
25	Synthetic polymer	4.18	0.175	78	0.645	29	Accept	3.05	Apply	3.51	Guided response
26	Denture based polymer	4.48	0.194	75	0.710	19	Accept	3.48	Apply	3.92	Guided response
27	Denture lining materials	4.42	0.177	92	0.684	24	Accept	3.61	Apply	4.05	Mechanism
28	Dental ceramic	4.76	0.117	79	0.752	10	Accept	3.98	Apply	3.77	Guided response
29	Porcelain fused to metal	4.73	0.124	75	0.745	12	Accept	3.97	Apply	3.77	Guided response
30	Emerging dental biomaterials (Bioceramic, Bioactive materials)	4.65	0.147	97	0.729	16	Accept	3.65	Apply	3.55	Guided response
31	Biomaterials for regenerative dentistry	4.61	0.156	95	0.723	18	Accept	3.42	Apply	3.40	Guided response
32	Materials in digital dentistry (3-D printing and computer-aided design and manufacturing)	4.44	0.178	92	0.687	22	Accept	3.26	Apply	3.29	Guided response
33	Material testing and characterisation technologies	4.05	0.149	80	0.648	28	Accept	2.92	Understand	3.00	Guided response
34	Dental implant materials	4.47	0.194	89	0.694	21	Accept	3.34	Apply	3.29	Guided response
35	Endodontic materials	4.92	0.045	92	0.784	1	Accept	4.19	Analyse	4.31	Mechanism
36	Temporary restorative materials	4.79	0.084	85	0.768	6	Accept	4.11	Analyse	4.45	Mechanism

Table 2 displays the mean scores for cognitive and psychomotor levels according to Bloom’s and Simpson’s taxonomies, respectively. Seventeen topics for dental materials science were deemed to be suitable for students at the cognitive level of “Apply” (C3), while 10 topics were deemed suitable at the cognitive level of “Understand” (C2) and the remaining nine topics were considered appropriate to attain a higher cognitive level of “Analyse” (C4). In general, experts did not consider the need for dental students to attain the lowest cognitive level of “Remember” (C1) or achieve higher cognitive levels of “Evaluate” (C5) and “Create” (C6). On the other hand, sixteen topics were deemed suitable for dental students to achieve the psychomotor level of “Guided response” (P3). Eight topics were considered suitable for a psychomotor level of “Set” (P2), whereas the remaining 11 topics were deemed appropriate to reach a higher psychomotor level of “Mechanism” (P4). Based on the mean scores, experts agreed that the lowest psychomotor levels of “Perception” (P1) and highest levels of “Complex Overt response” (P5), “Adaptation” (P6), and “Origination” (P7) are not required for undergraduate students.

## Discussion

The present study employed Fuzzy Delphi method to gather experts’ consensus in determining the relevant dental materials science topics for undergraduate curricula and their appropriate cognitive and psychomotor levels for each topic. The findings of the present study included 33 dental materials science topics that Malaysian dental students should master before graduating from their undergraduate dental programs. Moreover, Fuzzy Delphi method offers a reliable quantitative approach as compared to conventional Delphi method as each expert would encounter some degrees of uncertainty regarding a particular variable, which is referred to as the “grey area” [17]. Hence, a fuzzy Delphi method is used to mitigate the “grey area” impact and guarantee robust analysis [20]. In addition, the intervals and ambiguous meaning constraints of the Likert scale can be avoided using fuzzy Delphi mathematical analysis. The fuzzy Delphi method, which is regarded as a reliable and valid measurement tool, has been used in several studies to gather expert opinions in the field of dentistry [11, 21]. In the present study, a variety of opinions are combined to complement one another’s shortcomings to arrive at the desired outcome. The current established undergraduate dental materials science framework may be regarded as a model that was developed and consented by experts with minimal prejudice [20].

From the initial lists, three topics were excluded from the present study, namely “Dental amalgam”, “Direct gold filling” and “Investment and refractory die”. Although dental amalgams have been used as restorative materials for more than 150 years, the International Association for Dental Research has recommended that they should be phased out by 2024, apart from circumstances where no other dental restorative materials are available [22]. The Minamata Convention on Mercury, a global agreement aiming at preventing anthropogenic emissions and releases of mercury, has also issued a declaration on the phase-down of dental amalgam [23]. This could be another reason why experts in the present study could not reach a consensus on accepting “dental amalgam”. Furthermore, the Malaysian Dental Council clarified its viewpoint in a position statement that supports the Minamata Convention on Mercury and the FDI-World Dental Federation in their efforts to gradually phase-out the use of dental amalgam [24].

Apart from dental amalgam, “Direct gold filling” was also removed from the list based on the current finding. Historically, gold alloy has been favoured as a direct restorative material because of its superior wear characteristics [25]. However, new restorative materials including composite resins and glass ionomer cement have gradually replaced it owing to the metallic component that diminished aesthetics and translucency. Patients prefer tooth-coloured restorations in contemporary dentistry because they appear natural and are aesthetically pleasing. Moreover, gold restorative materials were noted to be time-consuming and expensive (which costs 8 to 9 times more than dental amalgam), rendering their popularity to decline over the years [26]. Furthermore, “Investment and refractory die” was excluded from the list possibly due to the nature of the topic as it is more relevant to dental technologists that focuses on dental laboratory work [27]. Thus, it is reasonable to presume that the majority of experts believe that this topic has little or no relevance to the dental clinical setting.

Fuzzy Delphi method can also be used as a content validation process to rank the significance of relevant dental materials science topics and exclude the unacceptable ones based on expert opinions [17]. Based on the present finding, the top-ranking topic was “Endodontic materials”, followed by “Dental composite resin”. Modern dental materials are at the cutting edge of endodontics, which primarily focuses on the preservation of the pulp and the teeth [28]. Due to the exceptional biological properties and mineralization potential of recently developed bioceramic-based endodontic materials, dental practitioners will now have to keep abreast with the most advanced materials on the market. Therefore, it is crucial that dental students should possess a solid theoretical knowledge base and exceptional clinical abilities in dealing with endodontic materials to deliver endodontic therapy and keep up with the most recently developed bioceramic endodontic materials. It is also essential to draw attention to the materials employed in “Endodontic materials” for minimally invasive or preventative endodontics, such as advanced calcium silicate or bioactive glass materials, which eliminates the need for more extensive and invasive endodontic therapy [29].

Since the advent of adhesive dentistry, dental composite resins have undergone a significant transformation and gradually replaced dental amalgam as the choice of permanent restorative materials [30, 31]. In addition, direct composite restoration is gaining vast acceptance among patients as it can closely match the natural tooth colour, making “Direct composite resin” the second highest ranked topic. Since dental composite resins require adhesive materials to bond to the tooth structure, it is not a surprise that the “Principle of adhesion: acid etch and bonding agent” was ranked third in the present study. Due to the demanding goal of creating long-lasting bonding to dentine utilizing resin monomers, dental adhesives have witnessed significant changes in their chemistry and combination of components over the past 40 years [32]. Indisputably, the long-term prognosis of dental composite resin is more predictable if there is a good adherence to tooth structures.

Nevertheless, it is imperative that dental students should have adequate cognitive and psychomotor competencies on the fundamental principles of dental materials as Malaysia is shifting its undergraduate dental curricula into competency-based dental programs which is in line with the national competency statement. There is a noticeable trend where topics in dental materials science related to metal and alloys were ranked at a lower cognitive and psychomotor level, whereas topics related to restorative and endodontic dental materials like “Dental composite resin”, “Glass ionomer cement”, “Dental cements”, “Endodontic materials”, and “Temporary restorative materials” were concurred to reach a higher cognitive and psychomotor level by experts. One can postulate that such a trend may be the result of experts believing that dental students should be more competent at selecting and working with dental materials used in clinics rather than those frequently utilized in dental laboratories.

With regards to the revised competency statement relevant to dental materials science course, dental students are required to justify the selection of dental materials based on the science and applications, and be able to manipulate commonly used dental materials, which correspond respectively to Bloom’s cognitive level C4 (Analyse) and Simpson’s psychomotor level P5 (Complex overt response). However, many topics for dental materials science were deemed to be suitable for students at the cognitive level of “Apply” (C3) and psychomotor level of “Guided response” (P3). It is unclear if experts generally do not anticipate students to attain higher levels of cognitive and psychomotor competence. A proper planning is required while designing and developing undergraduate dental curricula, as it will have a tremendous impact on the competencies attained by future dental practitioners. Nevertheless, the present findings can be adopted by other Malaysian public and private dental institutions in tailoring their existing undergraduate dental programs with better alignment with the learning outcomes and competencies based on the revised national competency statement. Plus, the present findings can serve as a baseline standard and be modified to meet the unique educational requirements of various countries.

The present study is the first of its kind to establish a consensus among experts in selecting relevant dental materials science topics. The advantage of the present study is that it involved a high response rate which can strengthen the reliability of the resulting consensus and reflects how experts perceived the importance of such a study [33]. The validity of the current study was also further improved by assembling a representative panel of experts from various expertise and affiliations. Hence, the findings of the present study may be used as a guide in other nations where there is a rising emphasis on enhancing the curriculum for dental materials science. Owing to fewer iterations, the fuzzy Delphi method asserts to be more beneficial and reuqires less time than traditional Delphi method [34]. However, one of the limitations of the present study was that, due to delayed responses, experts were reminded repeatedly to provide their responses, which might lead to emotional bias [17]. Furthermore, the investigators were unable to monitor the experts when they were answering the questionnaire online. Thus, there might be a possible communication or discussion among the participants, which may have resulted in responses that were not entirely independent [35]. Although Fuzzy Delphi method is best suited for addressing complex problems with a high degree of uncertainty, it might not be the optimum approach for dealing with quantitative objectives that have a set of limited or well-defined parameters [34]. In other words, fuzzy Delphi is a qualitative method that relies on subjective judgments rather than objective data. Despite having experts of similar expertise, some may have extensive knowledge of certain dental materials science topics, while vice versa for the others. This can lead to the possibility of bias and inconsistency in the results obtained [36]. Next, the proposed relevant dental materials science topics for undergraduate dental curricula may be viewed as a prototype since it has not been implemented and evaluated among Malaysian dental students. Thus, further studies are warranted to ascertain its applicability and effectiveness in undergraduate dental programs by developing measurable learning outcomes and incorporating innovative pedagogy as well as assessment criteria for each topic.

## Conclusion

The present study demonstrates the use of Fuzzy Delph method to gather experts’ consensus with minimal prejudice in developing content for dental curriculum. Thirty-three topics were identified as important topics and their competency levels were determined. “Endodontic materials” was ranked as the most significant topic with majority of the topics were deemed adequate at the cognitive level of “Apply” and psychomotor level of “Guided response”. Nonetheless, the selected relevant dental materials science topics still require additional construct validation and testing to ensure their applicability and effectiveness in undergraduate dental programs across the nation. Therefore, the next steps of the curriculum development would be developing corresponding learning outcomes, pedagogy, and assessments for these topics, followed by the implementation across dental schools in the country.

## Data Availability

All data generated or analysed during this study are included in this published article.

## Abbreviations

MDC: Malaysian Dental Council
AIMST: Asian Institute of Medicine, Science and Technology University
